# Diagnostic Performance of McMonnies Questionnaire as a Screening Survey for Dry Eye: A Multicenter Analysis

**DOI:** 10.1155/2016/6210853

**Published:** 2016-05-15

**Authors:** Yuxin Guo, Rongmei Peng, Kang Feng, Jing Hong

**Affiliations:** Department of Ophthalmology, Peking University Third Hospital, 49 North Garden Road, Haidian District, Beijing 100191, China

## Abstract

*Purpose*. To evaluate the diagnostic performance of the McMonnies questionnaire as a screening survey for dry eye in Chinese outpatients.* Methods*. The questionnaire was self-administered by 27,999 patients with dry eye symptoms. A thorough ophthalmic examination including tear break-up time (TBUT), fluorescein staining, and Schirmer I test was completed to make a clinical diagnosis of dry eye. Reliability, validity, and accuracy of the McMonnies questionnaire were assessed.* Results*. The McMonnies questionnaire showed poor internal consistency (Cronbach *α* = 0.37), but excellent validity as the scores correlated with TBUT (Spearman test, *r* = −0.322, *P* < 0.001) and Schirmer I test (Spearman's test, *r* = −0.370, *P* < 0.001), and significantly differed between the dry eye and control groups (2-sample* t*-test, *t* = 69.51, *P* < 0.001). The area under the receiver-operating characteristics (ROC) curve (AUC) was 0.729, suggesting moderate accuracy in identifying dry eye and non-dry eye patients. However, the AUCs varied significantly in different gender and age subgroups (*z* test, *P* < 0.001), as the discriminating ability declined with age. Analysis of the ROC curves also revealed that different cut-off points should be employed for each subgroup to achieve the same level of accuracy.* Conclusions*. The McMonnies questionnaire demonstrates moderate diagnostic value, and different cut-off points should be selected for various study populations.

## 1. Introduction

Dry eye is a multifactorial disease of the tears and ocular surface that results in symptoms of discomfort, visual disturbance, and tear film instability with potential damage to the ocular surface [1]. Dry eye could be either a separated clinical entity or a condition associated with other systemic or ocular surface diseases. Currently, it belongs to the most frequently encountered ocular problems in clinical practice.

As the definition of dry eye is still under constant revision, the lack of a “gold standard” for diagnosis challenges ophthalmologists worldwide. The International Dry Eye Workshop recommended confirming the diagnosis of dry eye based on a combination of symptoms and objective clinical tests [2]. Accordingly, the Chinese diagnostic criteria of dry eye were published by the Corneal Disease Study Group of Chinese Ophthalmological Society in 2013, offering a more defined standard [3].

The subjective symptoms of dry eye, along with certain risk factors, were advised to be screened by validated questionnaires [2]. As one of the most long-standing instruments, the McMonnies questionnaire is widely used in numerous prevalence studies [4–6] and clinical trials [7–9]. It contains 14 questions that revolve around the risk factors of dry eye, including age, gender, previous dry eye treatments, dry eye-related symptoms (both primary and secondary to environmental triggers), and systemic conditions associated with dry eye (dryness of mucous membranes, arthritis, thyroid disease, and medication use) [10].

Previous analyses [11–14] revealed varying values of sensitivity (34%–98%) and specificity (36%–97%) for the McMonnies questionnaire. Moreover, this instrument was originally devised from a sample of Australian women aged above 45 years with or without keratoconjunctivitis sicca syndrome [10, 11]. Subsequent studies [12–14] evaluating the reliability and validity of the instrument continued to focus on non-Asian populations. Thus, it is anticipated that variations in the diagnostic efficacy are likely to occur when applying the instrument to a Chinese cohort.

Therefore, we carried out a study in multiple ophthalmological centers across China to investigate the diagnostic performance of the McMonnies questionnaire as a screening survey for dry eye in Chinese outpatients.

## 2. Method

### 2.1. Patient Sample

The study was carried out in 94 ophthalmological centers, distributed in 45 cities, 23 provinces across China. Consecutive outpatients in general eye clinics were enrolled from July to November, 2013, if they presented with one or more of the following chief complaints: dryness, grittiness, burning sensation, tiredness, soreness, and visual disturbance. Participants are excluded if they exhibited any active infection of the eye, evidence of ocular chemical or thermal burn, ocular surgeries within 6 months before the screening, and pregnancy or lactation. Informed consents were obtained for each patient at each clinical site. The research was approved by Peking University Third Hospital Medical Ethics Committee and consistent with the tenets of the Declaration of Helsinki.

### 2.2. Assessment of Dry Eye

The McMonnies questionnaire, translated into Mandarin in advance, was self-administered by all participants. Then, each patient would be assessed by an ophthalmologist. The examining doctor had no knowledge of the results of the completed questionnaires. All subjects were required to remove their contact lens and discontinue any artificial tears for at least 2 hours before the assessment. Ophthalmic examinations were conducted in the following order:

1. *Inquiry of Medical History*. Information on ophthalmic and systemic disease was collected.
2. *Tear Break-Up Time (TBUT)*. A standard fluorescein stripe was moistened and used to lightly touch the inferior palpebral conjunctiva. The patient would be asked to blink several times. Under cobalt blue light of a slit-lamp, the time interval between the last blink and the appearance of the first desiccation spot would be recorded as TBUT.
3. *Keratoconjunctival Staining*. After TBUT test, any fluorescein staining of the corneas and interpalpebral conjunctiva was also recorded.
4. *Schirmer I Test*. Without anesthesia, a precalibrated standard stripe was placed in the lateral one-third of each lower fornix for 5 minutes. During this time, the patients were instructed to look downward or gently close their eyes. The length of the wetting was measured after removing the stripe.
5. *Slit-Lamp Exam*. Eyelid margins, including meibomian gland orifices and secretions, were evaluated under slit-lamp for pathological changes.

Test results of the more severely affected eye were recorded for further analyses. Diagnoses were established according to the Chinese diagnostic criteria of dry eye [3]: (1) presence of dry eye symptoms (dryness, grittiness, burning sensation, tiredness, soreness, or visual disturbance), with TBUT ≤ 5 s or Schirmer I test ≤ 5 mm/5 min; (2) presence of dry eye symptoms, with 5 s < TBUT ≤ 10 s or 5 mm/5 min < Schirmer I test ≤ 10 mm/5 min, accompanied by positive keratoconjunctival staining with fluorescein. Subjects conformed to either of the two criteria were clinically diagnosed with dry eye; otherwise they were classified as non-dry eye (control).

### 2.3. Statistical Analyses

Data analyses were performed using Statistical Package for the Social Sciences software, version 22.0 (SPSS Inc., Chicago, IL). A *P* value less than 0.05 was considered statistically significant, and 95% confidence intervals (CIs) were tabulated.

#### 2.3.1. Factor Analysis

The Kaiser-Meyer-Olkin measure was first calculated to test the degree of common variance, an assessment of whether the sample is adequate for factor analysis. Any value below 0.50 is interpreted as “unacceptable” for factor analysis. The Bartlett test of sphericity was also conducted to determine whether the items were sufficiently intercorrelated for factor analysis.

After weighing the adequacy, factor analysis with varimax rotation was performed to find out whether the items of the McMonnies questionnaire tend to cluster into certain domains.

#### 2.3.2. Reliability

The internal reliability of the McMonnies questionnaire was evaluated by Cronbach *α*. Generally, an *α* value greater than 0.70 is acceptable to indicate that the items of the instrument are measuring the same thing. It should be noted that the Cronbach *α* coefficient is an index dependent on the number of items in an instrument. Therefore, the average interitem correlation was also calculated, which is not affected by the number of items.

Due to the large sample size of our study, all participants only completed the questionnaire once. So we were unable to assess the test-retest reliability in this study.

#### 2.3.3. Validity

Concurrent validity was assessed by examining the correlation between scores of the questionnaire and 2 quantitative dry eye test results (i.e., TBUT and the Schirmer I test) using Spearman's rank correlation. Discriminant validity was evaluated using 2-sample* t*-test to determine the differences in scores between the dry eye and the control group.

#### 2.3.4. Accuracy

In order to maximize the diagnostic efficacy of the McMonnies questionnaire, receiver-operating characteristics (ROC) curves of both the entire sample and different gender and age groups were generated. ROC curves express the diagnostic accuracy of a test variable by plotting the sensitivity of the test against the specificity at all possible thresholds. This method was employed to select the most appropriate cut-off point for our study population.

The area under the ROC curve (AUC) is an index for diagnostic value: 0.5 means no discrimination between the affected and the control groups, while 1.0 indicates perfect discrimination. We compared the ROC curves of different gender and age groups using* z* tests to see if the diagnostic performance is compromised when applying the instrument to certain subpopulations.

We also summarized the positive likelihood ratio (LR+) of the McMonnies questionnaire in different groups, which is a measure that indicates how much the odds of the disease increase when a test is positive.

## 3. Results

### 3.1. Study Population

This study recruited 31,124 outpatients from the ophthalmology departments of 94 tertiary hospitals, distributed in 45 cities, 23 provinces across China. Among these participants, 27,999 (90.0%) have completed the questionnaire and all the dry eye tests. The demographics of the study population are listed in Table 1. The majority of our sample (44.6%) belonged to the age group of 25–45 years, whereas participants less than 25 years old made up the smallest portion (22.2%). Females accounted for 51.0% (14,280) of the sample. The overall prevalence of dry eye according to the Chinese diagnostic criteria was 58.8% (16,468), with the remaining 41.2% (11,531) classified as the control group. Prevalence of dry eye in each gender/age subgroup is also revealed in Table 1.

### 3.2. Factor Analysis

The Kaiser-Meyer-Olkin measure of sampling adequacy was 0.62, and the Bartlett test of sphericity was significant (*P* < 0.001), indicating sufficiency for factor analysis. Exploratory factor analysis of the McMonnies questionnaire revealed four potential factors, explaining only 47% of the cumulative variance. Such low communalities suggested that the extracted factors were insufficient to represent each item. Moreover, none of the factor loadings seemed to provide any logical meaning. For instance, one factor was composed of the question regarding a history of thyroid abnormality and the question about sleeping with eyes partly open. Therefore, the attempt to further analyze the questionnaire by domain was denied.

### 3.3. Reliability

The Cronbach *α* based on standardized items for the McMonnies questionnaire was 0.37, indicating poor internal consistency. Similarly, the average interitem correlation was 0.046 (range, −0.132 to 0.328), implying that each item refers to relatively independent aspects of the instrument's objective (i.e., screening dry eye).

### 3.4. Validity

The correlations between the scores of the McMonnies questionnaire and both dry eye test results were significant. The Spearman coefficient for the questionnaire and TBUT was −0.322 (*P* < 0.001) and −0.370 (*P* < 0.001) for Schirmer I test. Also, the mean scores were 17.27 ± 5.33 for the dry eye group and 12.82 ± 5.21 for the control group, respectively. The scores of the two groups were statistically different from each other (2-sample* t*-test, *t* = 69.51, *P* < 0.001).

### 3.5. Accuracy

**Figure 1:**
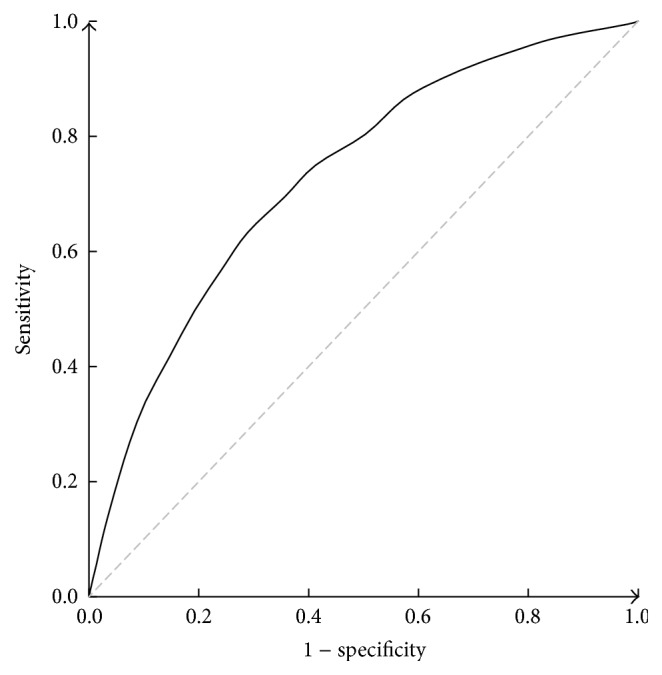
The overall ROC curve of the McMonnies questionnaire for screening dry eye.

**Figure 2:**
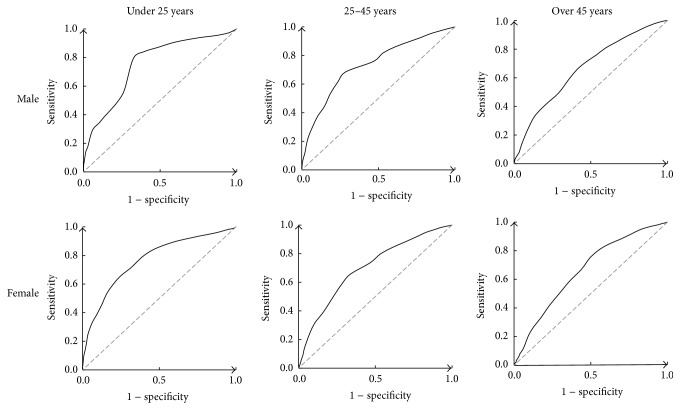
ROC curves of the McMonnies questionnaire in various gender and age groups. Note the relatively rounded curves (towards the upper left corner of the diagram) in younger subgroups, indicating good diagnostic performance.

The ROC curves of the McMonnies questionnaire in the entire sample as well as in various gender/age subgroups are shown in Figures 1 and 2. The sensitivity, specificity, and LR+ values at a cut-off point of 14.5 as recommended [12] are listed in Table 2, along with the AUCs of each group. The overall AUC of McMonnies questionnaire is 0.729 (95% CI, 0.723–0.735), indicating moderate discrimination. All AUCs of each group were significantly different from that of the entire sample (*z* test, *P* < 0.001), as the discriminating ability declined with age. Likewise, sensitivity, specificity, and LR+ all varied considerably among these cohorts.

Since the McMonnies questionnaire is used mainly as a screening method for dry eye, it is appropriate to maximize the sensitivity value, so as to avoid missed diagnosis. Accordingly, alternative thresholds were introduced for the instrument (Table 3). We also select separate cut-off points for each gender/age subpopulation with the highest specificity after setting the sensitivity at a value above 0.80, that is, those with the sensitivity values most approximate to 0.80 (Table 4).

## 4. Discussion

To our knowledge, this is the first multicenter study with a large sample investigating the diagnostic efficacy of the McMonnies questionnaire. Our results suggest that the instrument shows poor internal consistency, excellent validity, and moderate discriminating ability as a screening survey for dry eye in Chinese outpatients.

The McMonnies questionnaire was initially developed by reviewing literature, and scores of each item were tabulated based on clinical experience [10]. Several reports evaluating the diagnostic efficacy of the questionnaire have been published since then. McMonnies and Ho [11] tested the instrument in 100 women aged above 45 years with or without keratoconjunctivitis sicca and achieved 98% sensitivity and 97% specificity. The results were deemed biased, because they were derived from the same sample from which the cut-off value was determined. So they reassessed on an independent sample of 50 women with Sjögren syndrome and 124 normal controls, all over 45 years of age, and found a sensitivity of 92% and specificity of 93% with a weighted-scale algorithm [12]. Still, the data were affected by spectrum bias, since the severity of the disease in the study population was highly selective. Later on, Nichols et al. [14] reported a sensitivity and specificity of 82% and 36%, respectively, by identifying various degrees of dry eye severity in a sample without normal controls. Moreover, differences in the diagnostic criteria of dry eye used in these studies made it challenging to compare the results.

Nonetheless, certain psychometric properties of the McMonnies questionnaire can be compared with other studies, because they are irrelevant to diagnostic criteria. In our study, the questionnaire showed poor internal consistency as indicated by the Cronbach *α* and average interitem correlation. Such low internal reliability implies that each item of the instrument measures rather independent aspects of dry eye. This is also why the items failed to cluster into any logical domains by factor analysis. These were consistent with the results of Nichols et al. [14]. The authors inferred that the poor internal consistency would undermine the power associated with statistical significance tests, when comparing the scores between different groups or over time. Similar conclusions have been made using Rasch analysis [15], suggesting that the McMonnies questionnaire does not function as a measure.

Unlike previous studies, the instrument was found to have fine validity in our study population. The scores not only differed significantly between the dry eye and control groups but also strongly correlated with the results of TBUT and Schirmer I test. Several prevalence studies [16, 17] have indicated poor correlations between dry eye symptoms and objective clinical tests. Even so, we would argue that the McMonnies questionnaire is comprised of many aspects of dry eye rather than symptoms. It is possible that the objective test results are correlated with some unknown factors such as age, gender, or secondary symptoms caused by environmental triggers. After all, the Schirmer I test without anesthesia is technically a stimulus to the subjects' eyes. Besides, Hong et al. [18] reported that the Schirmer I test values were correlated with age in a Chinese cohort, while TBUT results were not. Differences in the ethnicity of the study populations may also contribute, which is beyond the scope of our study.

The McMonnies questionnaire showed moderate accuracy in screening dry eye. Further analysis of the ROC curves revealed varying discriminating abilities among different gender and age subgroups, as the AUCs decreased with age. This is a bit surprising, since the instrument was originally developed and adjusted with subjects over 45 years old [11]. Again, variations in experimental samples and criteria used for disease diagnosis should be taken into account here. The differences in diagnostic efficacy among each subgroup are substantial, especially when applied with the same threshold of 14.5 (Table 2). It even resulted with some LR+ values lower than 1.0, which meant that the odds of dry eye actually reduced after the scores of the questionnaire were deemed positive.

Therefore, we believe it is necessary to assign separate cut-off values for different gender and age subpopulation. As a screening method for dry eye, the McMonnies questionnaire is extremely cost-effective as it could be conducted in a self-administered manner by patients. The sensitivity values are suggested to be maximized to avoid missed diagnosis. This is particularly appropriate when the patients could be further assessed with routine ophthalmic examinations to reach a final diagnosis in clinics. Based on these reasons, we consider a sensitivity value over 0.80 acceptable for the screening purpose. The diagnostic performances of each subgroup showed less diversity with newly proposed cut-off points as depicted in Table 4. Nevertheless, we need to stress that these cut-off points were derived from selected Chinese outpatients with at least one of the typical dry eye complaints. Further studies are required to assess these proposed cut-off points for their efficacy on independent samples.

## 5. Conclusion

Our data suggest that the McMonnies questionnaire demonstrate poor internal consistency, fine validity, and moderate accuracy as a screening survey for dry eye in Chinese outpatients. It is recommend to become a routine process for dry eye diagnosis in the clinical practice of ophthalmologists. However, different cut-off points should be selected for various subpopulations.

## Figures and Tables

**Table 1:** Demographics of the study population and prevalence of dry eye in each gender/age group.

Gender/age	Sample size	Prevalence (%)
Overall	27999	58.8
Male		
Under 25 years	3433	51.1
25–45 years	5690	52.7
Over 45 years	4596	59.0
Female		
Under 25 years	2793	45.2
25–45 years	6784	62.8
Over 45 years	4703	73.9

**Table 2:** Sensitivities, specificities, LR+, and AUCs of the McMonnies questionnaire in different gender/age groups at a recommended cut-off point of 14.5^*∗*^.

Gender/age	Sensitivity (%)	Specificity (%)	LR+	AUC^#^
Overall	69.5	64.3	1.95	0.729 (0.723, 0.735)
Male				
Under 25 years	44.3	18.6	0.54	0.752 (0.736, 0.769)
25–45 years	61.7	23.7	0.81	0.734 (0.721, 0.747)
Over 45 years	71.4	47.4	1.36	0.665 (0.649, 0.681)
Female				
Under 25 years	53.3	15.7	0.63	0.767 (0.749, 0.784)
25–45 years	73.6	46.6	1.38	0.699 (0.686, 0.711)
Over 45 years	88.2	69.7	2.91	0.661 (0.643, 0.679)

^*∗*^LR+: positive likelihood ratio; AUC: area under the receiver-operating characteristics curves.

^#^Data presented as mean AUC (95% confidence interval).

**Table 3:** Alternative cut-off points for the McMonnies questionnaire.

Cut-off points	Sensitivity (%)	Specificity (%)	LR+
9.5	92.8	28.9	1.30
10.5	90.0	35.7	1.40
11.5	86.4	42.7	1.51
12.5	80.3	49.7	1.60
13.5	75.0	58.7	1.82
14.5	69.5	64.3	1.95
15.5	63.3	71.1	2.19
16.5	57.0	75.7	2.35

**Table 4:** Sensitivities, specificities, and LR+ of the McMonnies questionnaire in different gender/age groups at newly proposed cut-off points^*∗*^.

Gender/age	Proposed cut-off points^#^	Sensitivity (%)	Specificity (%)	LR+
Overall	12.5	80.3	49.7	1.60
Male				
Under 25 years	10.5	84.0	60.5	2.13
25–45 years	11.5	81.7	47.0	1.54
Over 45 years	12.5	80.7	40.5	1.36
Female				
Under 25 years	9.5	84.2	53.6	1.81
25–45 years	13.5	80.1	46.3	1.49
Over 45 years	16.5	81.2	43.8	1.44

^*∗*^LR+: positive likelihood ratio.

^#^Proposed cut-off points of each group are those with the highest specificity after setting the sensitivity at a value above 0.80.
